# Dr. J. J. Birmingham

**Published:** 1903-12

**Authors:** 


Dr. J. J. Birmingham, of Buffalo, a graduate of the University of Buffalo in 1881, died at his home, October 7, 1903, aged 50 years.

